# The expression of MUC5AC in patients with rhinosinusitis: A systematic review and meta‐analysis

**DOI:** 10.1002/clt2.70003

**Published:** 2024-10-31

**Authors:** Yitao Li

**Affiliations:** ^1^ Department of Otolaryngology Head and Neck Surgery First Hospital of Hainan Medical College Haikou China

**Keywords:** expression, meta‐analysis, MUC5AC, rhinosinusitis, systematic review

## Abstract

**Background:**

To understand the connection between Muc5AC expression and the likelihood of rhinosinusitis, with the goal of providing insights into its prospective use as a biomarker.

**Methods:**

We searched PubMed, Embase, Cochrane Library, China National Knowledge Infrastructure, and Wanfang databases for studies up to November 2023 to conduct a literature review. After screening and quality assessment, eligible studies meeting the criteria were included. Muc5AC expression and rhinosinusitis association was analyzed by STATA 14.0.

**Results:**

Including weighted mean difference and 95% confidence interval, were reported. The meta‐analysis included 16 studies with 1448 rhinosinusitis patients. MUC5AC expression was significantly up‐regulated in both chronic rhinosinusitis with nasal polyps (CRSwNP; WMD: 0.52; 95% CI: 0.41–0.63) and chronic rhinosinusitis without nasal polyps (CRSsNP; WMD: 0.42; 95% CI: 0.28–0.56) patients compared to controls. IHC positive area analysis corroborated these findings, with elevated MUC5AC levels in CRSwNP (WMD: 25.61; 95% CI: 22.41–28.81) and CRSsNP (WMD: 39.74; 95% CI: 25.6–53.88) patients. Subgroup analysis based on tissue type (nasal tissue fluid and sinus mucosa) consistently supported the overall results.

**Conclusion:**

Our meta‐analysis robustly demonstrates a significant association between elevated MUC5AC expression and rhinosinusitis risk. This finding underscores the potential of MUC5AC as a molecular marker, providing valuable insights for future research and potential therapeutic interventions in rhinosinusitis management.

**Systematic review registration:**

CRD42024518932.

## 1 INTRODUCTION

Chronic rhinosinusitis (CRS) is a commonly encountered otolaryngological disease, exhibiting a global prevalence.^1^ Two phenotypes^2^, ^3^, ^4^ characterize CRS based on tissue remodeling characteristics: chronic rhinosinusitis with nasal polyps (CRSwNP) and chronic rhinosinusitis without nasal polyps (CRSsNP).^5^ CRS is not restricted to a particular age range, and its morbidity rate rises with age. In China, the current morbidity rate of CRS ranges from 2% to 8%,^6^, ^7^ with an annual increase of 0.3% in the number of CRS patients.^8^ The current understanding of CRS pathogenesis is inconclusive, as emphasized by the lack of clarity in existing research.^9^

Several research directions have gained prominence in recent years, shedding light on potential contributors to CRS pathogenesis. Immunological factors, including aberrant inflammatory responses within the sinonasal mucosa, have been a focal point of investigation.^10^ Studies have identified elevated levels of pro‐inflammatory cytokines such as interleukin‐1β (IL‐1β), tumor necrosis factor‐α (TNF‐α), and interleukin‐6 (IL‐6), in the nasal secretions of CRS patients, pointing toward an inflammatory milieu.^4^, ^11^ Moreover, the role of innate and adaptive immune responses in CRS has been a subject of intense scrutiny. Research has implicated immune cells such as mast cells, eosinophils, and T lymphocytes in the perpetuation of sinonasal inflammation.^12^ Identification of distinct immune endotypes within CRS has further highlighted the need for personalized therapeutic approaches tailored to specific immunological profiles.^13^ Genetic factors have also emerged as crucial players in CRS pathogenesis. Investigations into the genetic basis of CRS susceptibility have revealed associations with polymorphisms in genes related to mucin production, immune regulation, and epithelial barrier function.^14^, ^15^ Understanding the genetic landscape of CRS not only aids in identifying individuals at higher risk but also offers insights into potential therapeutic targets.

Additionally, advances in molecular biology techniques have facilitated the exploration of gene expression patterns, uncovering potential biomarkers associated with CRS subtypes and disease severity.^13^ The discovery of distinct endotypes within CRS, characterized by specific immunological and molecular features, has opened new avenues for personalized medicine approaches.^16^ However, despite these strides, there remain significant gaps in our understanding of specific molecular players contributing to CRS pathogenesis, particularly in the context of mucin expression.

Expression of mucins in the human airways encompasses MUC2, MUC4, MUC5AC, and MUC5B. MUC5AC, a gene encoding mucin 5AC, emerges as the primary component of airway mucus among these mucins, and the elasticity and viscosity of airway mucus are largely determined by high‐density mucin glycoproteins.^17^ Mucins are large glycoproteins crucial for maintaining the integrity of the mucous layer lining the respiratory epithelium.^18^ Of the various mucin genes, MUC5AC has drawn attention due to its involvement in mucociliary clearance and potential dysregulation in inflammatory respiratory conditions. Various studies have explored mucin hypersecretion in CRS,^19^, ^20^ but the molecular mechanisms underpinning mucus hypersecretion and the overexpression of MUC5AC are not fully elucidated.

The relevance of MUC5AC in the context of CRS lies in its potential to serve as a molecular marker reflective of the underlying inflammatory processes. Given the diverse nature of CRS subtypes and the heterogeneity in clinical presentation, a focused examination of MUC5AC expression may shed light on shared molecular pathways or distinct profiles associated with specific subtypes. This, in turn, could inform diagnostic strategies, guide treatment decisions, and pave the way for targeted therapeutic interventions aimed at modulating mucin expression.

The aim of this systematic review and meta‐analysis was to bridge the existing knowledge gaps regarding the association between MUC5AC expression and CRS. By consolidating data from diverse studies, we seek to provide a comprehensive overview of the current state of evidence, explore potential variations across CRS subtypes, and assess the robustness of the association.

## 2 MATERIALS AND METHODS

This review was reported according to the PRISMA guideline and registered at the PROSPERO (https://www.crd.york.ac.uk/prospero/) as CRD42024518932.

### 2.1 Search strategy

Researchers utilized the search terms “MUC5AC,” “rhinosinusitis,” and “expression” to conduct literature searches in PubMed, Embase, Cochrane Library, China National Knowledge Infrastructure (CNKI), and Wanfang Database (Wanfang). The detailed search strategies used in the study's comprehensive search are outlined in Additional file 1. Inclusion criteria encompassed all studies published in full text without language restrictions. The search period extended from the establishment date of each database to November 2023.

### 2.2 Inclusion and exclusion criteria

Inclusion criteria: (1) Included patients diagnosed with rhinosinusitis, both CRSwNP and CRSsNP; (2) Get tested for MUC5AC levels by IHC or ELISA or omics; (3) Compared with healthy people/non‐rhinosinusitis; (4) The endpoint indicators are MUC5AC concentration and MUC5AC positive area. Exclusion criteria were as follows: (1) Animal experiments and case reports; (2) Duplicate publications; (3) Studies lacking full‐text access.

### 2.3 Data acquisition and quality evaluation

Two researchers undertook a review of potentially eligible studies and data collection. Disagreements were deliberated with a third researcher until consensus was attained. The following information was extracted from each of the included studies: first author, publication year, country, sample size, age, study design, diagnosis, female (%), comparison, tissue, and outcomes. Study quality was appraised using the Newcastle Ottawa Scale (NOS),^21^ with scores ranging from 0 to nine points. Literature was characterized as high‐quality if the total score was ≥7, and literature with a total score ≥6 was considered eligible for inclusion.

### 2.4 Statistical analysis

The data were analyzed using STATA 14.0,^22^ and forest plots illustrating continuous data were generated with the Weighted Mean Difference (WMD) and 95% confidence interval.^23^ Heterogeneity was examined using the *I* ^2^ statistic, with significance considered when *I* ^2^ > 50%. Random‐effects models were implemented in the presence of significant heterogeneity.^24^ A sensitivity analysis was undertaken to examine the potential impact of individual study results on the overall effect size when there was notable inter‐study heterogeneity.^25^ Employing subgroup analyses, we investigated potential sources of heterogeneity. Funnel plots and Egger's test were utilized to assess publication bias,^26^ and Trim and Fill analysis^27^ was implemented in instances of identified publication bias.

## 3 RESULTS

### 3.1 Study selection and characteristics

**FIGURE 1:**
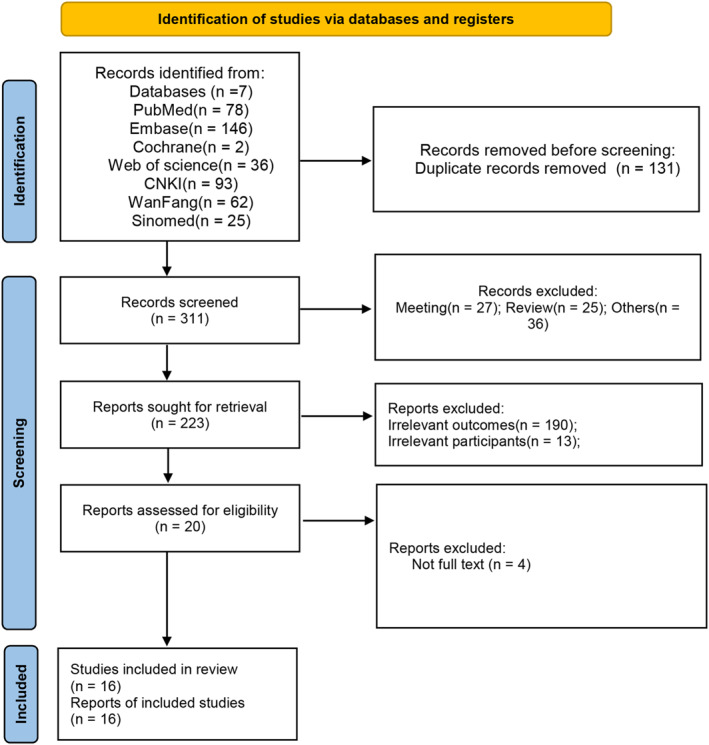
PRISMA flow chart of literature selection.

After the initial literature search (Figure 1), a total of 442 articles were identified, and 311 articles remained following duplicate removal. Screening of titles and abstracts resulted in the exclusion of 291 studies due to irrelevance or being animal studies, with 20 studies undergoing further examination through full‐text evaluation. The absence of full text led to the exclusion of four studies (*n* = 4). In the final meta‐analysis, 16 studies^19^, ^28^, ^29^, ^30^, ^31^, ^32^, ^33^, ^34^, ^35^, ^36^, ^37^, ^38^, ^39^, ^40^, ^41^, ^42^ were included, involving a total of 1448 subjects (Figure 1). Table 1 provides the essential characteristics of the included studies. Since 2006, a total of 16 articles, all cohort studies, have been included. The study population was recruited from three countries, predominantly China, with two articles each from England and the United States. Varied sample sizes were observed across studies, ranging from 13 to 240 participants. The methodological quality assessment, illustrated in Table S2 using the NOS scale with a score range of 0–9 stars, indicated high quality for each study.

**TABLE 1:** Characteristics of the studies included in this meta‐analysis.

Study	Country	Study design	Diagnosis^a^	Sample size	Age	Female%	Comparison	Tissue	Outcomes
Yang 2016^30^	China	Cohort study	1	65 (CRSwNP/CRSsNP/HC:25/25/15)	37.32	53.85	CRS versus HC	Sinus mucosa	IHC positive area
Chen 2023^21^	China	Cohort study	/	172 (CRSwNP/CRSsNP/HC:60/60/52)	41.79	51.74	CRS versus HC	Nasal tissue fluid	Expression level
Pena 2015^33^	USA	Cohort study	2	13 (CRS/non‐CRS:7/6)	12.71	46.15	CRS versus non‐CRS	Sinus mucosa	IHC positive area
Viswanathan 2016^34^	England	Cohort study	3	21 (CRS/transnasal hypophysectomy:14/7)	/	/	CRS versus non‐CRS	Nasal tissue fluid	Expression level
Zhang 2018^31^	China	Cohort study	4	124 (CRS/septum rectifying:62/62)	43.79	44.35	CRS versus non‐CRS	Nasal polyps	IHC positive area
Xu 2016^29^	China	Cohort study	/	150 (CRS/non‐CRS:75/75)	43.18	49.33	CRS versus non‐CRS	Nasal polyps	IHC positive area
Tang 2023^26^	China	Cohort study	5	110 (CRSwNP/CRSsNP/septum rectifying:40/40/30)	34.26	40	CRS versus non‐CRS	Sinus mucosa	IHC positive area, expression level
YangH 2016^24^	China	Cohort study	/	80 (CRS/HC:40/40)	36.55	51.25	CRS versus HC	Sinus mucosa	Expression level
Yin 2016^28^	China	Cohort study	6	185 (CRSwNP/CRSsNP/non‐CRS:25/25/15)	33.96	45.41	CRS versus non‐CRS	Sinus mucosa	IHC positive area, expression level
Wang 2021^27^	China	Cohort study	1	45 (CRSwNP/CRSsNP/non‐CRS:15/15/15)	45.72	46.67	CRS versus non‐CRS	Sinus mucosa	IHC positive area
Yu 2017^32^	China	Cohort study	6	45 (CRSwNP/CRSsNP/non‐CRS:15/15/15)	40.47	33.33	CRS versus non‐CRS	Sinus mucosa	IHC positive area
Luo 2015^35^	China	Cohort study	1	40 (CRSwNP/CRSsNP/traumatic neuropathy:15/15/10)	33.13	42.5	CRS versus non‐CRS	Uncinate processes	IHC positive area
Ding 2007^12^	China	Cohort study	/	20 (CRS/non‐CRS:10/10)	/	/	CRS versus non‐CRS	Sinus mucosa	IHC positive area
He 2017^23^	China	Cohort study	6	240 (CRSwNP/CRSsNP/HC:80/80/80)	36.77	61.25	CRS versus HC	Sinus mucosa	Expression level
Shi 2022^25^	China	Cohort study	6	108 (CRS/non‐CRS:80/28)	41.06	40.74	CRS versus non‐CRS	Sinus mucosa	Expression level
Ding 2006^22^	China	Cohort study	7	30 (CRSwNP/CRSsNP/non‐CRS:10/10/10)	/	/	CRS versus non‐CRS	Sinus mucosa	IHC positive area

Abbreviations: CRS, chronic rhinosinusitis; CRSwNP, chronic rhinosinusitis with nasal polyps; CRSsNP, chronic rhinosinusitis without nasal polyps.

^a^ :1: European position paper on rhinosinusitis and nasal polyps 2012 (EPOS 2012). 2: Persistent symptoms for more than 3 months, despite antimicrobial and topical steroid therapies. 3: Nasal endoscopy and sinus CT scans. 4: Pathology. 5: EPOS 2020. 6: Guidelines for diagnosis and treatment of chronic rhinosinusitis (2012). 7: Guidelines for diagnosis and treatment of chronic rhinosinusitis (1997).

### 3.2 MUC5AC expression analysis

**FIGURE 2:**
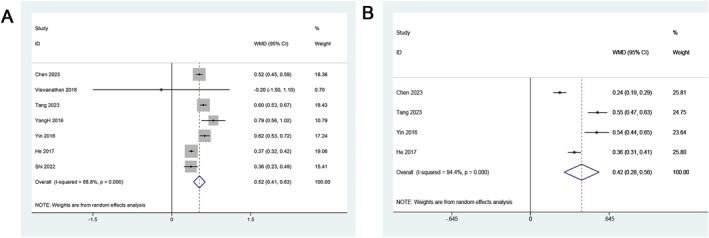
Forest plots of MUC5AC expression between patients with and without CRS. (A) CRSwNP; (B) CRSsNP. CRS, Chronic rhinosinusitis; CRSwNP, chronic rhinosinusitis with nasal polyps; CRSsNP, chronic rhinosinusitis without nasal polyps.

For CRSwNP patients, the WMD was 0.52 (95% CI: 0.41–0.63), indicating a statistically significant increase in MUC5AC expression. However, substantial heterogeneity was observed (*I* ^2^ = 88.8%, *p* < 0.001), suggesting potential variability across studies (Figure 2A). Similarly, for CRSsNP patients, the WMD was 0.42 (95% CI: 0.28–0.56), signifying a significant up‐regulation of MUC5AC. Heterogeneity remained high (*I* ^2^ = 94.4%, *p* < 0.001), emphasizing the need for cautious interpretation (Figure 2B).

### 3.3 IHC positive area analysis

**FIGURE 3:**
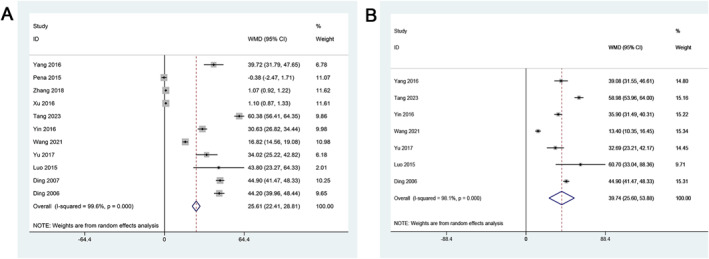
Forest plots of the IHC positive area for MUC5AC expression between patients with and without CRS. (A) CRSwNP; (B) CRSsNP. CRS, Chronic rhinosinusitis; CRSwNP, chronic rhinosinusitis with nasal polyps; CRSsNP, chronic rhinosinusitis without nasal polyps.

For CRSwNP patients, the WMD was 25.61 (95% CI: 22.41–28.81), indicating a significant elevation in MUC5AC protein expression. However, heterogeneity was exceptionally high (*I* ^2^ = 99.6%, *p* < 0.001), emphasizing the potential diversity in protein‐level assessments (Figure 3A). In CRSsNP patients, the WMD was 39.74 (95% CI: 25.6–53.88), further underscoring a substantial up‐regulation of MUC5AC protein expression. Heterogeneity persisted (*I* ^2^ = 98.1%, *p* < 0.001), warranting cautious interpretation (Figure 3B).

### 3.4 Subgroup analysis

The subgroup analysis based on tissue type, including nasal tissue fluid and sinus mucosa, aimed to explore whether MUC5AC expression patterns varied across different anatomical compartments. Subgroup analysis based on tissue type consistently supported the overall results (Figure S1A–D).

### 3.5 Sensitivity analysis

**FIGURE 4:**
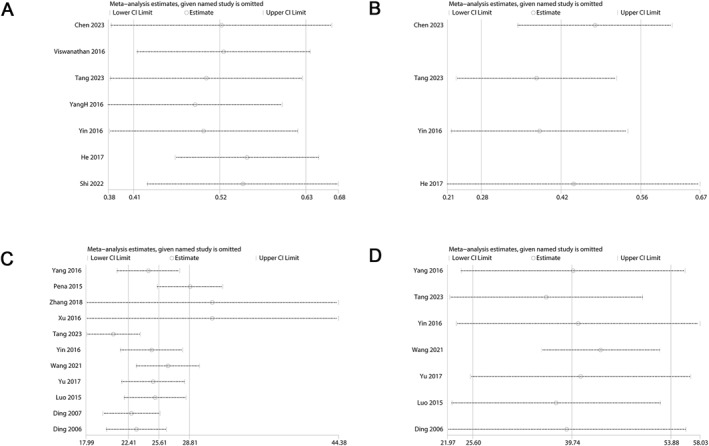
Sensitivity analysis examining the influence of individual studies on pooled results. (A) MUC5AC expression for CRSwNP; (B) MUC5AC expression for CRSsNP; (C) IHC positive area analysis for CRSwNP; (D) IHC positive area analysis for CRSsNP. CRSwNP, chronic rhinosinusitis with nasal polyps; CRSsNP, chronic rhinosinusitis without nasal polyps.

Substantial heterogeneity was observed, prompting sensitivity analyses to assess the impact of individual studies on overall results. The exclusion of a single study did not substantially alter the findings, indicating the robustness of the observed associations (Figure 4A–D).

### 3.6 Publication bias

**FIGURE 5:**
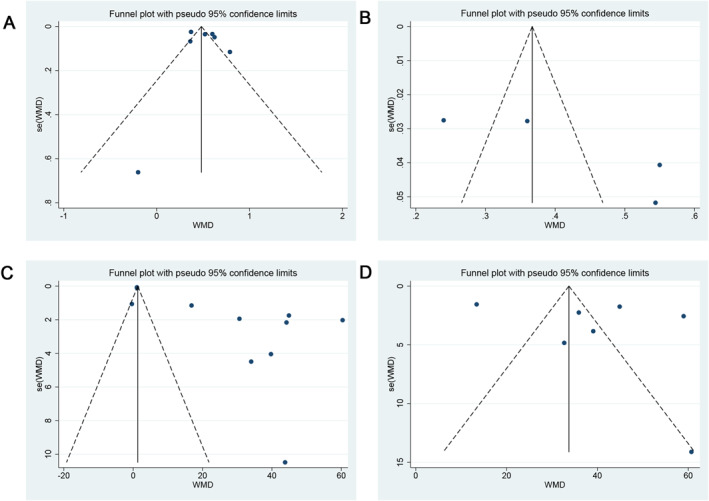
Funnel plot for publication bias. (A) MUC5AC expression for CRSwNP; (B) MUC5AC expression for CRSsNP; (C) IHC positive area analysis for CRSwNP; (D) IHC positive area analysis for CRSsNP. CRSwNP, chronic rhinosinusitis with nasal polyps; CRSsNP, chronic rhinosinusitis without nasal polyps.

The use of funnel plots, along with Begg's and Egger's tests, for investigating publication bias revealed no significant publication bias in the analysis of MUC5AC expression for CRSwNP patients (*p* = 1.000 and *p* = 0.558 according to Begg's and Egger's tests, respectively; Figure 5A), CRSsNP patients (*p* = 0.308 and *p* = 0.148 according to Begg's and Egger's tests, respectively; Figure 5B), and IHC positive area analysis for CRSsNP patients (*p* = 0.764 and *p* = 0.456 according to Begg's and Egger's tests, respectively; Figure 5C). However, there was a notable publication bias identified in the analysis of MUC5AC expression and IHC positive area for CRSwNP patients (*p* = 0.35 and *p* = 0.002 according to Begg's and Egger's tests, respectively; Figure 5D). Furthermore, in the “trim and fill” analysis for IHC positive area in CRSwNP patients, five missing studies were estimated, resulting in a pooled odds ratio estimate of 3.348 (95% CI: 2.961, 3.785; Figure S2).

## 4 DISCUSSION

Our systematic review and meta‐analysis aimed to comprehensively investigate the association between MUC5AC expression and rhinosinusitis. The synthesis of data from 16 studies, encompassing 1448 patients with rhinosinusitis, consistently revealed a significant up‐regulation of MUC5AC in both CRSwNP and CRSsNP patients compared with controls. This association holds true for both protein‐level and IHC positive area assessments, reinforcing the robustness and consistency of MUC5AC up‐regulation at the molecular level in the context of rhinosinusitis.

The origins of CRS and nasal polyposis remain poorly elucidated.^43^ Mucins, including MUC5AC, are integral components of the protective mucous layer that lines the respiratory epithelium. One primary function of this mucous layer is to maintain mucosal integrity by acting as a physical barrier. MUC5AC, produced by goblet cells in the respiratory epithelium, contributes significantly to the formation of this protective layer.^17^ This barrier serves as the first line of defense against inhaled pathogens, irritants, and particulate matter. In chronic inflammatory upper airway diseases such as CRS, the mucosal integrity may be compromised, leading to increased susceptibility to infections and environmental insults.^14^ Despite this, a prevalent pathological change in chronic inflammatory upper airway diseases involves secretory cell hyperplasia and increased mucus secretion.^44^ Despite several recent studies^28^, ^32^, ^33^, ^34^ investigating the expression of MUC5AC at both mRNA and protein levels in the nasal epithelium, there is currently a gap in the literature regarding meta‐analyses comparing the expression of mucin genes and proteins in the epithelium between CRS and control groups. Our findings align with and extend the existing literature on MUC5AC expression in rhinosinusitis. While individual studies have reported increased MUC5AC levels in rhinosinusitis patients, our meta‐analysis provides a comprehensive synthesis of existing evidence, offering a more nuanced and statistically robust understanding of this association.^45^ Previous reviews and studies^46^, ^47^ have often focused on specific aspects of mucin dysregulation in rhinosinusitis, but our meta‐analysis aggregates data from diverse studies to provide a comprehensive overview.

The underlying mechanisms driving MUC5AC dysregulation in rhinosinusitis warrant exploration.^10^ The inflammatory milieu characteristic of rhinosinusitis, marked by immune cell infiltration, cytokine release, and tissue remodeling, likely contributes to the observed up‐regulation of MUC5AC.^48^ Recent studies have highlighted the pivotal role of immune cell infiltration in the regulation of mucin gene expression, including MUC5AC.^49^ In rhinosinusitis, immune cells, particularly neutrophils, eosinophils, and lymphocytes, infiltrate the sinonasal mucosa in response to ongoing inflammation.^50^ These immune cells release a plethora of inflammatory mediators, including cytokines and chemokines, that collectively orchestrate the local immune response. The activation of signaling pathways within resident epithelial cells, including goblet cells responsible for MUC5AC production, is a consequence of this immune cell infiltration.^51^ Pro‐inflammatory cytokines such as interleukin‐1β (IL‐1β), TNF‐α, and interleukin‐13 (IL‐13), have been implicated in mucin gene expression regulation. These cytokines can activate signaling pathways that lead to increased mucin production, including MUC5AC.

Furthermore, interactions between epithelial cells and infiltrating immune cells, such as eosinophils and mast cells, may play a role in mucin dysregulation.^52^ Eosinophils, for example, are known to release granule proteins that can stimulate mucin secretion. Investigating the specific molecular pathways activated in response to inflammation in different rhinosinusitis subtypes could unveil key targets for therapeutic interventions.

While our meta‐analysis provides valuable insights, several limitations should be considered when interpreting the results. The presence of substantial heterogeneity, especially in protein‐level analyses, indicates potential variability across studies. Variations in patient characteristics, disease severity, and laboratory techniques contribute to this heterogeneity. Subgroup analyses were conducted to explore potential sources of heterogeneity, but inherent differences in study designs may still impact the overall results. Publication bias, as indicated by asymmetrical funnel plots and significant Egger's test results, is another consideration. Studies with smaller sample sizes and less favorable outcomes may be underrepresented in the published literature, potentially influencing the overall effect estimates. Despite these limitations, the inclusion of studies with diverse methodologies and the stability of results in sensitivity analyses enhance the robustness of our findings. Additionally, the cross‐sectional nature of the included studies limits our ability to establish causality. Longitudinal studies tracking MUC5AC expression changes over time in rhinosinusitis patients would provide more insight into the dynamic nature of mucin dysregulation in the course of the disease. Exploring the functional implications of increased MUC5AC expression in rhinosinusitis is crucial for understanding its role in disease pathogenesis. This includes investigating the impact of elevated MUC5AC on mucus rheology, ciliary function, and microbial interactions within the sinonasal cavity. Moreover, elucidating the signaling pathways and immune cell interactions involved in mucin dysregulation could unveil potential therapeutic targets for intervention. Therapeutically, targeting MUC5AC dysregulation could offer a novel approach to rhinosinusitis management. Strategies aimed at modulating mucin expression, such as targeting specific cytokine pathways or mucin‐stimulating receptors, could be explored. However, the potential downstream effects of altering mucin expression must be carefully considered as mucins play multifaceted roles in respiratory physiology.

## 5 CONCLUSION

In conclusion, our systematic review and meta‐analysis provide compelling evidence for the up‐regulation of MUC5AC expression in patients with rhinosinusitis. The consistency of findings across diverse study methodologies, rhinosinusitis subtypes, and anatomical compartments strengthens the reliability of our results. Elevated MUC5AC expression likely reflects an adaptive response to the inflammatory environment, emphasizing its potential as a molecular marker in rhinosinusitis pathogenesis.

## AUTHOR CONTRIBUTIONS

**Yitao Li**: Conceptualization; investigation; funding acquisition; writing—original draft; methodology; validation; visualization; software; formal analysis; project administration; data curation; supervision; resources; writing—review & editing.

## CONFLICT OF INTEREST STATEMENT

The authors declare that they have no conflicts of interest.

## CONSENT FOR PUBLICATION

Not applicable.

## CONSENT TO PARTICIPATE

Not applicable.

## Supporting information

Supporting Information S1

Table S1

Table S2

## Data Availability

All data generated or analyzed during this study are included in this published article.
