# Supplementary material for: The expression of MUC5AC in patients with rhinosinusitis: A systematic review and meta‐analysis
Source: Clin Transl Allergy. 2024 Oct 31;14(11):e70003. doi: 10.1002/clt2.70003 (PMC11527733; doi:10.1002/clt2.70003)
Supplement: Supplementary file 1 — Supporting Information S1 [file CLT2-14-e70003-s003.docx]

**
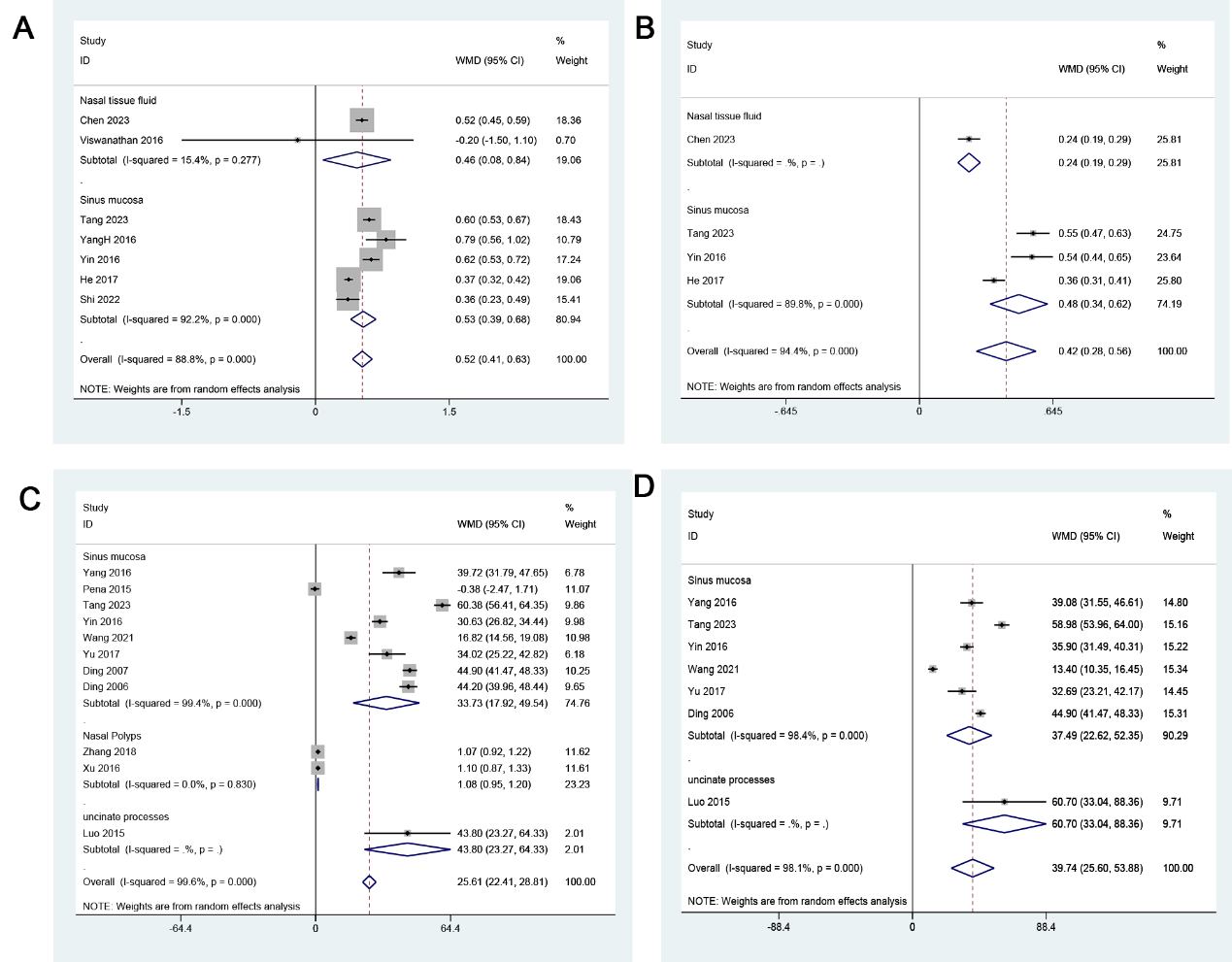
**

**Figure S1** Subgroup analysis of MUC5AC expression between patients with and without CRS. A) MUC5AC expression for CRSwNP; B) MUC5AC expression for CRSsNP; C) IHC positive area analysis for CRSwNP; D) IHC positive area analysis for CRSsNP.

**
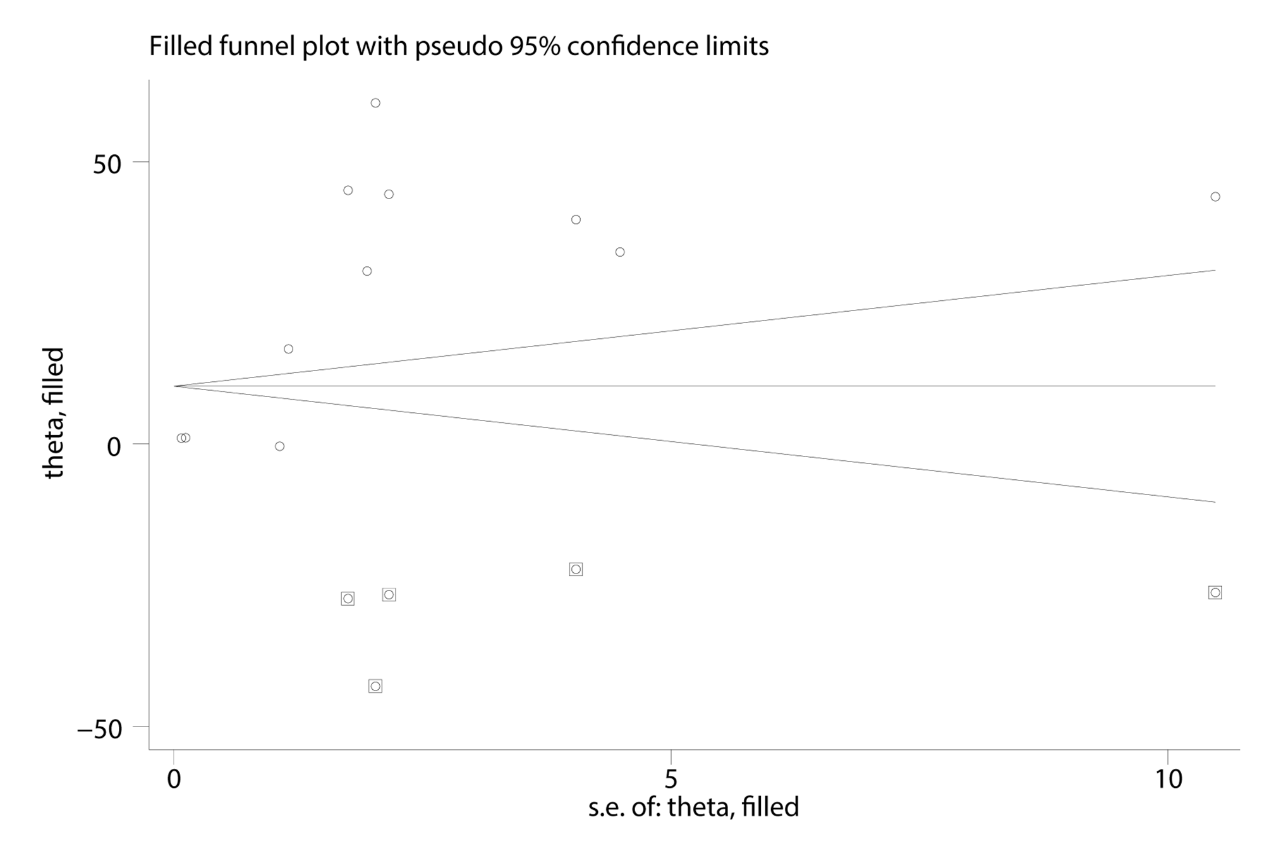
**

**Figure S2** the cut and fill method for IHC positive area for CRSwNP.

**Supplementary table title:**

**Table S1.** Search strategy

**Table S2.**Literature quality assessment based on the 9-star NOS.
