# Supplementary material for: The expression of MUC5AC in patients with rhinosinusitis: A systematic review and meta‐analysis
Source: Clin Transl Allergy. 2024 Oct 31;14(11):e70003. doi: 10.1002/clt2.70003 (PMC11527733; doi:10.1002/clt2.70003)
Supplement: Supplementary file 2 — Table S1 [file CLT2-14-e70003-s001.docx]

**Search strategy of PubMed**

| NO. | Search Details | Results |
| --- | --- | --- |
| #5 | (#1 OR #2) AND (#3 OR #4) | 78 |
| #4 | (((((Mucin 5C) OR (Mucin 5A)) OR (MUC5AC)) OR (MUC5AC mucin)) OR (MUC5AC protein)) OR (mucin 5AC) | 3,834 |
| #3 | "Mucin 5AC"[Mesh] | 1,769 |
| #2 | ((((((Sinusitides) OR (Sinus Infections)) OR (Sinus Infection)) OR (pansinusitis)) OR (sphenoid sinusitis)) OR (sphenoidal sinusitis)) OR (sinusitis) | 78,193 |
| #1 | "Sinusitis"[Mesh] | 23,484 |

**Search strategy of EMBASE**

| No. | Query | Results |
| --- | --- | --- |
| #5 | (#1 OR #2) AND (#3 OR #4) | 146 |
| #4 | 'mucin 5c':ti,ab,kw OR 'mucin 5a':ti,ab,kw OR 'muc5ac':ti,ab,kw OR 'muc5ac mucin':ti,ab,kw OR 'muc5ac protein':ti,ab,kw OR 'mucin 5ac':ti,ab,kw | 5130 |
| #3 | 'mucin 5ac'/exp | 5292 |
| #2 | 'sinusitides':ti,ab,kw OR 'sinus infections':ti,ab,kw OR 'sinus infection':ti,ab,kw OR 'pansinusitis':ti,ab,kw OR 'sphenoid sinusitis':ti,ab,kw OR 'sphenoidal sinusitis':ti,ab,kw OR 'sinusitis':ti,ab,kw | 29341 |
| #1 | 'sinusitis'/exp | 57960 |

**Search strategy of Cochrane Library**

| NO. | Search deatiles | Hits |
| --- | --- | --- |
| #1 | MeSH descriptor: [Sinusitis] explode all trees | 1430 |
| #2 | (Sinusitides):ti,ab,kw OR (Sinus Infections):ti,ab,kw OR (Sinus Infection):ti,ab,kw OR (pansinusitis):ti,ab,kw OR (sphenoid sinusitis):ti,ab,kw OR (sphenoidal sinusitis):ti,ab,kw OR (sinusitis):ti,ab,kw | 4181 |
| #3 | MeSH descriptor: [Mucin 5AC] explode all trees | 10 |
| #4 | (Mucin 5C):ti,ab,kw OR (Mucin 5A):ti,ab,kw OR (MUC5AC):ti,ab,kw OR (MUC5AC mucin):ti,ab,kw OR (MUC5AC protein):ti,ab,kw OR (mucin 5AC):ti,ab,kw | 69 |
| #5 | (#1 OR #2) AND (#3 OR #4) | 2 |

**Search strategy of CNKI（English version）**

| NO. | Search deatiles | Hits |
| --- | --- | --- |
| #1 | (((((TS=(Sinusitides) OR TS=(Sinus Infections)) OR TS=(Sinus Infection)) OR TS=(pansinusitis)) OR TS=(sphenoid sinusitis)) OR TS=(sphenoidal sinusitis)) OR TS=(sinusitis) | 24330 |
| #2 | ((((TS=(Mucin 5C) OR TS=(Mucin 5A)) OR TS=(MUC5AC)) OR TS=(MUC5AC mucin)) OR TS=(MUC5AC protein)) OR TS=(mucin 5AC) | 3913 |
| #3 | #2 AND #1 | 36 |

**Search strategy of CNKI（Chinese version）**

**(SU %= 'bidayan' OR SU %= 'bidayanxirou' OR SU %= 'huanongxingbidayan' OR SU %= 'bidayanzheng' OR SU %= 'manxingbidayan' OR SU %= 'bidayanzheng' OR SU %= 'fufaxingbidayan') AND (SU %= 'mucin' OR SU %= 'mucin5ac') 93**

**Search strategy of WanFang**

**(zhuti:(bidayan) or zhuti:(bidayanxirou) or zhuti:(huanongxingbidayan) or timinghuoguanjianzi:(bidayanzheng) or zhuti:(manxingbidayan) or zhuti:(bidayanzheng) or zhuti:(fufaxingbidayan)) and (zhuti:(mucin) or zhuti:(mucin5ac)) 62**

Search strategy of Sinomed

("mucin5ac"[changyongziduan:zhineng] OR "mucin"[changyongziduan:zhineng]) AND ("bidayan"[changyongziduan:zhineng] OR "bidayanxirou"[changyongziduan:zhineng] OR "huanongxingbidayan"[changyongziduan:zhineng] OR "bidayanzheng"[changyongziduan:zhineng] OR "manxingbidayan"[changyongziduan:zhineng] OR "bidayanzheng"[changyongziduan:zhineng] OR "fufaxingbidayan"[changyongziduan:zhineng]) 25
